# Gut microbiota in regulation of childhood bone growth

**DOI:** 10.1113/EP091620

**Published:** 2023-12-29

**Authors:** Julian C. Lui

**Affiliations:** ^1^ Section on Growth and Development National Institute of Child Health and Human Development Bethesda Maryland USA

**Keywords:** clinical trial, insulin‐like growth factor I, microbiome, probiotics, short stature

## Abstract

Childhood stunting and wasting, or decreased linear and ponderal growth associated with undernutrition, continue to be a major global public health challenge. Although many of the current therapeutic and dietary interventions have significantly reduced childhood mortality caused by undernutrition, there remain great inefficacies in improving childhood stunting. Longitudinal bone growth in children is governed by different genetic, nutritional and other environmental factors acting systemically on the endocrine system and locally at the growth plate. Recent studies have shown that this intricate interplay between nutritional and hormonal regulation of the growth plate could involve the gut microbiota, highlighting the importance of a holistic approach in tackling childhood undernutrition. In this review, I focus on the mechanistic insights provided by these recent advances in gut microbiota research and discuss ongoing development of microbiota‐based therapeutics in humans, which could be the missing link in solving undernutrition and childhood stunting.

## Abstract (highlights)

- **What is the topic of this review?**
  This review is about the mechanisms by which the gut microbiota regulates bone growth.
- **What advances does it highlight?**
  There is in vivo evidence for regulation of the gut microbiota on the growth hormone–insulin‐like growth factor I axis. An association exists between gut microbiota immaturity and undernutrition. Clinical trials of microbiota‐based therapeutics are underway.

## 1 INTRODUCTION

Human height, or its quantitative variability among individuals, is the cumulative result of hundreds or thousands of genes and their interactions with the environment. This is because childhood bone growth, as dictated by what happens inside the epiphyseal growth plates found near the ends of our long bones, is regulated by many different genes expressed locally at the growth plates and numerous endocrine signals, which are in turn influenced by nutrition and other environmental factors (Zhou & Lui, 2021). Consequently, genetic disorders affecting the growth plate (e.g., achondroplasia), disruption of the endocrine systems (e.g., growth hormone deficiency), medical conditions affecting nutritional intake (e.g., coeliac disease) and chronic undernutrition could all result in diminished bone growth and short stature in children (Lui, 2017). Human height is also strongly associated with level of education, economic productivity and life expectancy in the world population. Unfortunately, childhood stunting, which is defined by the World Health Organization as a height‐for‐age *z*‐score (HAZ) of less than two, relative to the international growth reference, is still commonly found in low‐ or middle‐income countries and remains a major global public health challenge. Despite our vastly improved understanding of the genetics of human stature in the general population and in various Mendelian disorders, current therapeutic interventions on undernutrition have only limited efficacies in improving anthropometric parameters, such as HAZ and weight‐for‐age *z*‐score (WAZ).

The term ‘gut microbiota’ refers to the community of microbes, including bacteria, archaea and eukarya, residing within the intestinal tract and engaging in a symbiotic relationship with the host (Jones et al., 2023). Consequently, the gut microbiota affects many aspects of human health, including nutritional intake, metabolism, the immune response and neurobehavioural development (Valdes et al., 2018). In the past decade, exciting research has demonstrated that the gut microbiota plays an important role in the hormonal and nutritional regulation of bone growth and body growth (de Vadder et al., 2021). In this review, I focus on the mechanistic insights provided by these recent advances and discuss ongoing development of microbiota‐based therapeutics for the treatment of undernutrition and childhood stunting.

## 2 REGULATION OF CHILDHOOD BONE GROWTH

### 2.1 Growth hormone–insulin‐like growth factor I axis and bone growth

**FIGURE 1:**
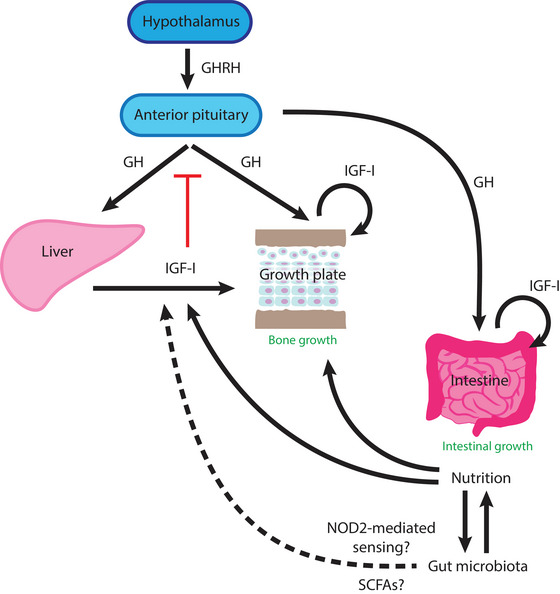
Regulation of bone growth by the GH/IGF‐I axis. Secretion of GH from the anterior pituitary is positively regulated by GHRH produced by the hypothalamus. Growth hormone stimulates production of IGF‐I in the liver, which then acts as an endocrine factor to stimulate bone growth at the growth plate. Growth hormone also stimulates local IGF‐I production in target tissues, such as the growth plate and the intestine, which acts as a paracrine/autocrine growth factor. Nutritional status positively regulates bone growth and maturation of the gut microbiota, which reciprocally promote nutritional intake. The gut microbiota also promotes bone growth, perhaps directly, by stimulating IGF‐I production. Possible mechanisms for such stimulation might involve SCFAs and NOD2‐mediated bacterial sensing pathways in the intestinal epithelial cells. Pointed arrows indicate stimulation; red blunted arrow indicates suppression. Abbreviations: GH, growth hormone; GHRH, growth hormone‐releasing hormone; IGF‐I, insulin‐like growth factor 1; NOD2, nucleotide‐binding oligomerization domain‐containing protein 2, SCFAs short‐chain fatty acids.

Childhood bone growth is governed by a complex interplay between different endocrine signals, including growth hormone (GH), insulin‐like growth factor I (IGF‐I), thyroid hormone, glucocorticoids and sex steroids, such as oestrogen. Instead of providing an extensive review of this topic, I focus on the GH/IGF‐I axis, which is by far the most important endocrine regulator of bone growth and has recently been indicated in gut microbiota interactions (Figure 1).

Secretion of GH in the anterior pituitary gland is stimulated by GH‐releasing hormone (GHRH) produced in the hypothalamus (Figure 1). Growth hormone then travels in the circulation as an endocrine factor to act on its target tissues, where the GH receptor is expressed, such as the liver, the intestine and the growth plate. Although GH is also known to have a direct growth‐promoting effect locally on its target tissue (Liu et al., 2017), the predominant function of GH is to activate the production of IGF‐I, which itself is a potent stimulator of growth. When GH‐induced IGF‐I production happens in the liver, the hepatic IGF‐I complexes with the IGF‐binding protein IGFBP3 and functions as an endocrine factor, acting on IGF‐I receptors and stimulating growth in many different tissues throughout the body (Yakar et al., 2018). When non‐hepatic IGF‐I is produced locally, such as in the growth plate, IGF‐I acts as an autocrine or paracrine factor to stimulate stem cell recruitment, proliferation and hypertrophic differentiation of chondrocytes (Lui et al., 2019), all of which are essential for driving bone elongation. In addition, GH‐induced local IGF‐I also supports epithelial stem cell proliferation and absorption of nutrients in the small intestine (Zheng et al., 2018). The importance of the GH/IGF‐I axis for body growth is demonstrated by pituitary adenomas that lead to elevated systemic GH and IGF‐I causing gigantism and acromegaly and, conversely, deficiency of GH leading to decreased IGF‐I and short stature. Similar to many other endocrine systems, a negative feedback loop exists between circulating IGF‐I levels and GH production in the pituitary. Therefore, in patients with GH insensitivity (also known as Laron syndrome) attributable to loss‐of‐function mutations of the GH receptor, IGF‐I production is diminished, leading to poor linear growth despite elevated GH levels caused by the lack of feedback inhibition. Interestingly, GH production in the pituitary is also regulated by several hormones related to energy metabolism and appetite. Somatostatin, which is produced in the hypothalamus and the gastrointestinal tract, suppresses GH production. In contrast, ghrelin and leptin, produced by the stomach and adipose tissue, respectively, both stimulate GH production.

### 2.2 Nutritional regulation of the GH/IGF‐I axis

Nutrition plays an important role in modulating the GH/IGF‐I axis of bone growth. During chronic undernutrition, GH receptor expression is downregulated in both the liver and the growth plate (Wu et al., 2013), thereby limiting the ability of circulating GH to induce both hepatic and peripheral IGF‐I production and leading to a state of GH insensitivity similar to that in Laron syndrome. Undernourished children therefore have decreased systemic IGF‐I but elevated GH levels, primarily owing to the lack of negative feedback from IGF‐I, in addition to elevated levels of hunger‐induced ghrelin (El‐Hodhod et al., 2009). Consequently, decreased IGF‐I signalling via the IGF‐I receptor can directly limit chondrocyte proliferation and hypertrophic differentiation in the growth plate (Lui et al., 2019). In addition, decreased IGF‐I and scarcity of essential amino acids during undernutrition can also suppress mechanistic target of rapamycin (mTOR) signalling, which, in turn, negatively regulates cell division and survival (Sancak et al., 2008).

### 2.3 Growth plate senescence and catch‐up growth

The rate of longitudinal growth is most rapid in the first 1000 days of life and gradually slows as we approach our final adult height. This growth deceleration is associated with the gradual decline in growth plate function, also known as growth plate senescence. Importantly, growth plate senescence is characterized by a gradual depletion of chondrogenic stem cells, decreasing chondrocyte proliferation and hypertrophy in the growth plate (Lui et al., 2011). Although growth plate senescence is generally associated with age, it appears not to be driven by age per se but instead depends on how much growth potential has been ‘used up’. In other words, chondrocytes in the growth plate appear to have a finite amount of growth potential, which is depleted gradually as more bone growth occurs, leading to the gradual decline in growth rate and the associated changes of senescence. This is supported by the fact that growth‐inhibiting conditions, such as undernutrition, can slow down growth plate senescence, allowing bone growth not only to resume but temporarily to accelerate faster than normal for chronological age once nutritional status improves, a clinical phenomenon known as catch‐up growth (Forcinito et al., 2011).

## 3 GUT MICROBIOTA AND BONE GROWTH

### 3.1 Effect of the gut microbiota on bone growth

In 2016, a couple of studies highlighted the importance of the gut microbiota in bone growth. Schwarzer et al. (2016) showed that germ‐free mice (lacking the whole microbiota, including that in the gut) have decreased body growth and decreased longitudinal bone growth compared with wild‐type mice. This growth deficit in germ‐free mice appears to be caused by the lack of the gut microbiota, because gut recolonization with *Lactobacillus plantarum* was able to rescue much of their growth deficiency. In a similar study by Yan et al. (2016), long‐term gut microbiota colonization in germ‐free mice improved bone formation and long bone length. Taken together, these two studies showed convincingly that the presence of the gut microbiota is beneficial, and perhaps essential, to normal bone growth.

### 3.2 Possible effect of the gut microbiota on IGF‐I

The molecular mechanisms by which the gut microbiota supports bone growth are not clear. A connection via the GH/IGF‐I axis has been proposed based on the observation that circulating IGF‐I and IGFBP3 levels were downregulated in the germ‐free mice (Schwarzer et al., 2016), which is reversed upon microbiota colonization (Schwarzer et al., 2016; Yan et al., 2016). One exciting possibility is that the gut microbiota specifically regulates the host GH/IGF‐I axis, such that, for example, hepatic IGF‐I production is stimulated by certain molecules or metabolites released by the microbes. However, another possible explanation is that the gut microbiota normally aids in macronutrient digestion (Oliphant & Allen‐Vercoe, 2019), which becomes less effective in germ‐free mice, causing a mild state of undernutrition. In that case, the observed decrease in IGF‐I and IGFBP3 with no change in GH is likely to represent GH insensitivity, which is one of the many hormonal changes induced by undernutrition. Importantly, these two explanations are not mutually exclusive and both could be true.

To test whether the gut microbiota regulates bone growth via the GH/IGF‐I axis, one could try to restore circulating IGF‐I in germ‐free mice to levels similar to the wild‐type mice and ask whether that alone is sufficient to restore bone growth fully. Injection of recombinant IGF‐I (for 10 days, twice daily, at 5 mg/kg) was able to improve bone growth in germ‐free mice but not to a statistically significant extent in wild‐type mice, suggesting that the growth deficit is driven by reduced IGF‐I in the germ‐free mice (Schwarzer et al., 2016). However, there are some important caveats to this interpretation. Injection of a high level of recombinant IGF‐I results in a higher than normal level of circulating IGF‐I (Gillespie et al., 1990), which is likely to improve bone growth regardless of whether or not the original growth deficit is IGF‐I dependent, similar to when GH treatment was typically used in children without GH deficiency. One could point to the promising observation that recombinant IGF‐I preferentially improved bone growth in germ‐free mice but not in wild‐type mice. However, because the germ‐free mice did not grow as well as the wild‐type mice before treatment, there was likely to be more growth potential remaining in the growth plate of the germ‐free mice, and therefore, recombinant IGF‐I might elicit a more prominent growth response (or catch‐up growth) owing to delayed growth plate senescence.

### 3.3 Possible regulatory mechanisms of the gut microbiota on IGF‐I

If indeed the gut microbiota regulates the GH/IGF‐I axis specifically, what could be the molecular signal by which the microbes stimulate IGF‐I secretion? One possibility is short‐chain fatty acids (SCFAs), which are major metabolites produced during fermentation of dietary fibre (Wong et al., 2006). Yan et al. (2016) showed that in mice in which the gut microbiota is depleted by broad‐spectrum antibiotics or vancomycin, the reduction in serum IGF‐I can be reversed by supplementation with SCFAs. The limitations of their findings were that long‐term bone growth was not assessed upon treatment with SCFAs, and they did not determine whether SCFAs alone could be used to stimulate IGF‐I and bone growth in germ‐free mice. The observed induction of IGF‐I by SCFAs has since been replicated by later studies (Czernik et al., 2021) and in systems other than bone growth, such as in prostate cancer (Matsushita et al., 2021).

If SCFAs were able to stimulate the GH/IGF‐I axis, it is unclear which signalling pathways could enable this. The SCFA receptors involved in IGF‐I production could be G‐coupled protein receptors (GPRs) 41 and 43, whose expression in bone could be induced by faecal microbiota transplantation (Xiao et al., 2022). Ghrelin has been proposed to allow SCFA‐induced modulation of the GH/IGF‐I axis because it can stimulate GH secretion in the pituitary. Interestingly, an acute increase in colonic SCFAs is negatively associated with the level of ghrelin in humans (Rahat‐Rozenbloom et al., 2017). However, ghrelin stimulates GH secretion rather than suppressing it, in which case the presence of the gut microbiota should induce SCFAs, thus lowering (rather than increasing) ghrelin, GH and IGF‐I production.

The signal by which the gut microbiota stimulates IGF‐I might not even be a metabolite. In fact, a recent study showed that bacterial cell walls isolated from *L. plantarum* were sufficient to stimulate IGF‐I and bone growth in mice (Schwarzer et al., 2023), suggesting that sensing of postbiotics by the host might be able to induce growth‐promoting metabolic and hormonal signals (Matos et al., 2017). This postbiotic sensing appears to be driven by innate immune receptor nucleotide‐binding oligomerization domain‐containing protein 2 (NOD2) expressed in the intestinal epithelial cells, because treatment with bacterial cell walls was unable to induce IGF‐I and improve bone growth in *Nod2*‐null mice (Schwarzer et al., 2023). Interestingly, the same study showed that NOD2‐activating ligands, such as muramyl dipeptide or the synthetic NOD2‐activating adjuvant mifamurtide, alone were sufficient to induce IGF‐I and bone growth, suggesting that NOD2 agonists could be a new class of therapeutic agents for improving childhood stunting.

### 3.4 Inflammatory cytokines and other possible mechanisms

Could the gut microbiota support bone growth by some molecular mechanisms other than those mentioned above? One potential mechanism could be by modulating the effect of inflammatory cytokines on bone growth (Sederquist et al., 2014). It is well established that chronic inflammatory diseases, such as inflammatory bowel disease, Crohn's disease, ulcerative colitis and juvenile idiopathic arthritis, negatively impact childhood bone growth (d'Angelo et al., 2021). Part of this growth impairment is attributable to undernutrition associated with these conditions and to the adverse side‐effects of the glucocorticoid therapy used for treatment. However, another major cause of growth inhibition comes from a local effect of cytokines, which are often elevated in inflammatory diseases. At a systemic level, pro‐inflammatory cytokines can inhibit bone growth by suppressing IGF‐I. For example, in mice overexpressing interleukin‐6 (IL‐6), body growth is significantly suppressed, with decreased IGF‐I and IGFBP3 but with normal levels of GH (de Benedetti et al., 2002). At a local level, the pro‐inflammatory cytokines tumor necrosis factor alpha (TNFα), interleukin‐1β (IL‐1β) and IL‐6 could all inhibit chondrocyte proliferation and hypertrophy while promoting apoptosis in the growth plate (Mårtensson et al., 2004).

The gut microbiota has been shown to influence circulating levels of pro‐inflammatory cytokines (Mizutani et al., 2022). A recent study by Webster et al. (2022) showed that serum IL‐1β and IL‐6 levels were correlated with the presence of certain bacterial strains in the gut microbiome. Mechanistically, butyrate, one of the SCFAs produced by the gut microbiota, has been shown to inhibit the inflammatory response elicit by lipopolysaccharides, TNFα and interleukins via GRP41 and GRP43, both in endothelial cells (Li et al., 2018) and in chondrocytes (Pirozzi et al., 2018), suggesting that the gut microbiota could stimulate bone growth by reducing inflammation. It would be interesting to test whether some of the growth‐promoting effect of the gut microbiota, including its effect on IGF‐I, is mediated by suppression of inflammation in vivo.

In addition to the GH/IGF‐I axis and inflammatory cytokines, the regulation of bone growth by the microbiome is likely to be multifactorial. Future studies on serum metabolomic analysis and transcriptome profiling of growth plate chondrocytes in germ‐free mice recolonized with different gut microbiotas and with various nutritional statuses will provide more comprehensive insights into the role of the gut microbiome and bone growth via other molecular mechanisms and signalling pathways.

## 4 GUT MICROBIOTA AS A THERAPEUTIC TARGET

### 4.1 Healthy maturation of the gut microbiota

The gut microbiota is established in newborns under the influence of multiple factors, including the birth mode, the maternal microbiota, breastfeeding and other environmental factors. During early infancy, the biodiversity of the gut microbiota remains narrow. For example, in breastfed infants, many of their gut microbiota are originated from the mother's breat milk oligosaccharide metabolism and originate from the mother's breast milk (Pannaraj et al., 2017). During the first year of life, the infant gut microbiota undergoes massive changes, shaped largely by nutritional availability and the increased dietary complexity. Gradually, a stable and phylogenetically diverse gut microbiota is established by 2–3 years of age (Koenig et al., 2011).

This succession and maturation of the gut microbiota plays an important role in the normal development and maintenance of various aspects of human health. In a study of healthy infants in Bangladesh, 24 age‐predictive bacterial taxa were identified, whose changes in abundance during the first 2 years of life could be used to define the process of normal gut microbiome maturation (Subramanian et al., 2014). Their findings serve as an important reference to evaluate the maturity of the gut microbiota, in the form of the microbiota‐for‐age *z*‐score, which is significantly correlated with the chronological age of children and with healthy growth phenotypes, such as WAZ and HAZ (Subramanian et al., 2014). A recent study showed that bacterial taxonomy in the gut microbiota might not necessarily predict the future growth trajectory; instead, the functional metagenomic features of the gut microbiome, which could still be taxa dependent, are better indicators for linear and ponderal growth and growth velocities (Robertson et al., 2023).

### 4.2 A vicious cycle of childhood stunting

**FIGURE 2:**
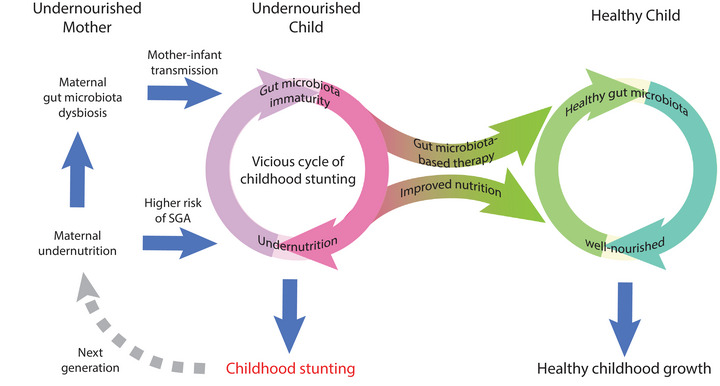
A vicious cycle of childhood stunting. Undernutrition in children could lead to gut microbiota immaturity, which itself could contribute to undernutrition and childhood stunting. This undernutrition cycle can also be intergenerational, because maternal size and maternal undernutrition are risk factors for SGA. Maternal undernutrition could also lead to maternal gut microbiota dysbiosis, which could be transmitted to the infant at birth. Improving nutritional intake by dietary intervention and microbiota‐based therapy might both be needed to escape this vicious cycle and achieve healthy childhood growth. Abbreviation: SGA, small for gestational age.

Persistent immaturity of the gut microbiota is associated with childhood undernutrition, which is particularly profound in children with severe acute malnutrition (SAM) (Subramanian et al., 2014). In children with SAM, nutritional interventions only partially improved their gut microbiota maturity and were completely ineffective in improving HAZ (Subramanian et al., 2014). Reciprocally, colonization of microbiota from undernourished children in gnotobiotic mice and pigs was sufficient to induce stunted bone growth and body growth (Gehrig et al., 2019; Wagner et al., 2016). Considering that the gut microbiota might also impact nutritional intake, these findings suggest a vicious cycle of childhood stunting, wherein undernutrition‐induced gut microbiota dysbiosis itself contributes to undernutrition and childhood stunting (Figure 2). This undernutrition cycle might also be intergenerational, because the maternal microbiota, which could be nutrition dependent, strongly influences early fetal growth, birth weight and the maturation of the microbiota of the infant (Gough et al., 2021). Furthermore, some evidence suggests that the negative impact of undernutrition on the gut microbiota could become harder to correct when it traverses across multiple generations (Sonnenburg et al., 2016). Therefore, consideration should be given to repairing the gut microbiome as part of the prevention and management of undernutrition in solving childhood stunting.

### 4.3 Therapeutic strategy for repairing the gut microbiome

**FIGURE 3:**
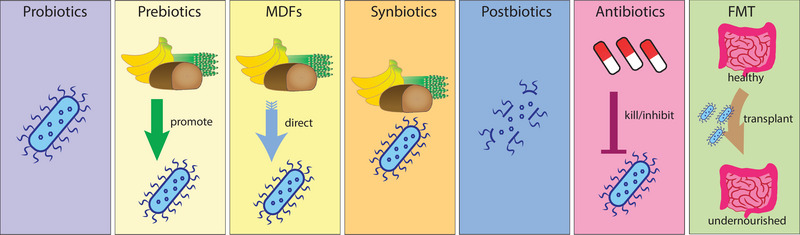
Different classes of microbiota‐based therapeutics. Probiotics are live microbes (usually in food) that contribute beneficially to the gut microbiota. Prebiotics are ingredients in food (such as fibre) that help to stimulate growth of gut microbiota. Microbiota‐directed food is specially formulated food that helps to shape or direct the growth of certain bacteria or improve microbiota maturation. Synbiotics are mixtures of probiotics and prebiotics. Postbiotics are materials left behind by probiotics, such as the bacterial cell wall. Antibiotics are antimicrobial substances that kill or inhibit microbes, which might help to reset microbiota equilibrium. Faecal microbiota transplant is a procedure that delivers mature gut microbiota from a healthy human donor stool to a recipient, such as an undernourished child with gut microbiota dysbiosis. Abbreviations: FMT, faecal microbiota transplant; MDFs, microbiota‐directed foods.

Microbiota‐based therapies (Figure 3) have shown some promising results in animal models. For example, a five‐species consortium of growth‐supporting bacteria was able to alleviate the growth deficiency in mice colonized with the gut microbiota from an undernourished donor (Blanton et al., 2016). In a similar study mentioned above, introduction of certain strain of *L. plantarum* (or simply isolated bacterial cell wall) could ameliorate growth phenotypes during undernutrition in mice (Schwarzer et al., 2023, 2016), providing a strong rationale for testing different approaches in clinical trials. Here, I highlighted the success stories of several types of microbiota‐based therapies tested in humans (Table 1).

**TABLE 1:** Recent clinical trials of microbiota‐based therapy.

Type	Subjects	Treatment	Phenotypic outcome	Reference
Probiotics/synbiotics	2‐ to 6‐month‐old infants with severe acute malnutrition (Bangladesh)	*Bifidobacterium infantis*, strain EVC001, alone or with HMO vs. placebo, once daily for 1 month, with 1 month follow‐up	Increased WAZ and MUAC in both the probiotic and synbiotic groups	Barratt et al. (2022)
MDFs	12‐ to 18‐month‐old children with moderate acute malnutrition (Bangladesh)	Microbiota‐directed food MDCF‐1, ‐2 and ‐3, vs. RUTF, twice daily for 4 weeks, with 2 weeks follow‐up	Improved MUAC and improved plasma proteomics (increased IGF‐I and IGFBP3)	Gehrig et al. (2019)
MDFs	12‐ to 18‐month‐old children with moderate acute malnutrition (Bangladesh)	Microbiota‐directed food MDCF‐2 vs. RUTF, twice daily for 3 months, with 1 month follow‐up	Improved weekly change of WHZ and WAZ; no improvement in MUAC	Chen et al. (2021)
Antibiotics	6‐ to 59‐month‐old children with severe acute malnutrition (Niger)	Amoxicillin (80 mg/kg), once daily for 7 days, vs. placebo, with 12 weeks follow‐up	Improved MUAC after 1 and 4 weeks, no improvement after 12 weeks	Isanaka et al. (2020, 2016)
Antibiotics	Secondary analysis of the Nigerian trial (Isanaka et al., 2016)	Amoxicillin (80 mg/kg), once daily for 7 days, vs. placebo, with 2 years follow‐up	Improved gut microbiota maturity, but growth was not assessed	Schwartz et al. (2023)

Abbreviations: HMO, human milk oligosaccharide; IGF‐I, insulin‐like growth factor 1; IGFBP3, IGF binding protein 3; MDCF, microbiota‐directed complementary food; MDF, microbiota‐directed food; MUAC, mid‐upper‐arm circumference; RUTF, ready‐to‐use therapeutic food; WAZ, weight‐for‐age *z*‐score; WHZ, weight‐for‐height *z*‐score.

### 4.4 Therapy with probiotics and synbiotics

Previously, two large clinical trials among children with SAM in Malawi (Kerac et al., 2009) and Uganda (Grenov et al., 2017) showed disappointing nutritional recovery after probiotic treatment. However, in a recently published single‐blind, randomized clinical trial called SYNERGIE (standing for SYNbiotic for Emergency Relief of Gut Instability and Enteropathy), weight gain was significantly improved by probiotics in 2‐ to 6‐month‐old SAM infants in Bangladesh.

*Bifidobacterium infantis*, which is commonly found in the gut microbiota of healthy breastfed infants but depleted in SAM infants, was used alone (probiotic arm) or in combination with the human milk oligosaccharide lacto‐*N*‐neotetraose (synbiotic arm) and compared with lactose (placebo arm). The study showed that after 1 month of daily administration of EVC001 (a commercially available strain of *B. infantis*), with or without human milk oligosaccharide, both WAZ and mid‐upper arm circumference (MUAC) were significantly improved versus placebo (probiotic, *n* = 20; symbiotic, *n* = 21; placebo, *n* = 21) (Barratt et al., 2022). A follow‐up study with a larger number of participants and longer recovery period might help to confirm the effectiveness of probiotic therapy and explain the discrepancy in efficacies between different trials.

### 4.5 Microbiome‐directed food

Similar to prebiotics, which are ingredients in food that promote growth of gut microbes, microbiome‐directed foods (MDFs) are specially designed formulations of food with the ability to boost representation of key growth‐promoting gut microbes and improve gut microbiota maturity. In an initial trial in 2019, children (mean age, 15.2 ± 2.1 months) in Bangladesh with SAM were treated with ready‐to‐use supplementary food (RUSF) or three formulations of MDFs (*n* = 14–17 per group) for 4 weeks, with a 2 week follow‐up (Gehrig et al., 2019). One of the formulations, MDCF‐2, significantly improved weight gain (measured by MUAC), with increased plasma levels of IGF‐I and IGFBP3 (Gehrig et al., 2019). In a subsequent trial (Chen et al., 2021), with a longer period of intervention (3 months) and focusing on MDCF‐2, the weekly change in WAZ and weight‐for‐height *z*‐score both improved in MDCF‐treated patients, compared with RUSF‐treated patients (MDCF‐2, *n* = 61; RUSF, *n* = 62). This effect appeared to be mediated by repair of the gut microbiota by the MDF, because bacterial taxa that are reported to be involved in normal gut microbiota maturation were significantly increased in the MDF‐treated group. The enrichment of these bacterial taxa was also positively correlated with the weight‐for‐height *z*‐score in these patients. However, none of these studies has provided long‐term follow‐up to assess the improvement in bone growth meaningfully, which would be extremely informative.

### 4.6 Conflicting effects of antibiotics

The World Health Organization recommends the use of a short course of antibiotics, such as amoxicillin, for outpatient management of uncomplicated SAM. However, antibiotics can also indiscriminately target both pathogenic and commensal bacteria, thus perturbing the gut microbiome ecosystem. It was previously shown that even short‐term use of antibiotics in preterm infants could have a potentially harmful and long‐lasting effect on the gut microbiota (Gasparrini et al., 2019). In a large clinical trial comparing 7 days of treatment with amoxicillin or placebo in Nigerian children with SAM, short‐term nutritional outcomes (measured by MUAC) were significantly improved after 1 month (Isanaka et al., 2016) but largely disappeared after 1 year (Isanaka et al., 2020). However, in a recently published secondary analysis of the Nigerian trial, Schwartz et al. (2023) focused on antibiotic resistance and the gut microbiota and found that although amoxicillin transiently increased antibiotic resistance genes and decreased the diversity of the gut microbiota, these changes largely subsided within 3 weeks. Surprisingly, 2 years after the initial treatment, the amoxicillin‐treated children had increased gut microbiome diversity and richness relative to placebo‐treated control children (Schwartz et al., 2023), suggesting an unexpected long‐term benefit that might outweigh the short‐term risks of antibiotic resistance. A follow‐up study on metabolic and anthropometric analysis will help to clarify whether or not antibiotic treatment has any long‐term benefit for bone growth.

## 5 CONCLUSION

A deeper understanding of the gut microbiota in general and in the context of the endocrine and nutritional regulation of bone growth has highlighted the importance of a holistic approach in solving undernutrition. In addition to the bare minimum of improving nutritional status, mitigation of gut microbiota dysbiosis, either by introducing growth‐stimulating bacterial strains or by promoting gut microbiota maturation, should be considered as coupling therapeutic strategies. As we continue to uncover the mechanistic links in host–microbe interactions, more clinical trials with larger sample sizes, more extensive follow‐up and for newer classes of microbiome‐based interventions are surely on the horizon. If the gut microbiota has been the missing link for childhood stunting all along, the stage could be finally set for public health policy‐makers to develop and implement effective treatment or even preventive care to solve childhood stunting once and for all.

## AUTHOR CONTRIBUTIONS

Sole author.

## CONFLICT OF INTEREST

The author declares no conflicts of interest.
